# Nosocomial Methemoglobinemia Resulting from Self-Administration of Benzocaine Spray

**DOI:** 10.1155/2015/685304

**Published:** 2015-10-28

**Authors:** Christopher Hoffman, Hawa Abubakar, Pramood Kalikiri, Michael Green

**Affiliations:** Drexel University College of Medicine, Hahnemann University Hospital, Philadelphia, PA 19102, USA

## Abstract

Methemoglobinemia is life-threatening and bears pathognomonic signs difficult to diagnose in real time. Local anesthetics are widely used and are known for eliciting this condition. We report a case of methemoglobinemia secondary to self-administered use of benzocaine spray. A 27-year-old woman was found to be in respiratory distress during postoperative recovery. After desaturation persisted, arterial blood gas yielded a methemoglobin level of 47%. The patient was successfully treated with intravenous methylene blue. Review of the events revealed self-administered doses of benzocaine spray to alleviate discomfort from a nasogastric tube. We review this case in detail in addition to discussing methemoglobinemia and its relevant biochemistry, pathophysiology, clinical presentation, and medical management. Given the recognized risk of methemoglobinemia associated with benzocaine use, we recommend its removal from the market in favor of safer alternatives.

## 1. Introduction

Methemoglobin arises when the ferrous (Fe2+) iron moiety of the heme group within hemoglobin is converted to the ferric (Fe3+) state. This occurs in the setting of oxidative stress and converts the heme group into a non-oxygen binding state. If allowed to persist in high concentration, significant hindrance of oxygen delivery to tissue occurs leading to hypoxia, lactic acidosis, and death [1, 2]. The red blood cell, as the primary oxygen carrier, is constantly exposed to oxidative stress with the concomitant risk of methemoglobinemia development.

Local anesthetics enhance methemoglobin formation as much as 1000-fold mainly through the oxidizing activity of their metabolites [3, 4]. Severity and frequency vary among anesthetics; a 2009 retrospective search of 242 reported episodes of methemoglobinemia yielded 159 cases with benzocaine, 68 cases with prilocaine, and 12 cases with lidocaine, among others [5]. The methemoglobinemia that arises leads to functional anemia and inadequate tissue oxygen delivery can result in symptoms observable even in the setting of modest increases in percent methemoglobin. Breakdown of symptom presentation to corresponding percentage methemoglobin values can be found in Table 1 [6].

Given the nonspecific nature of the presenting symptoms, diagnosis can be particularly challenging. If methemoglobinemia is allowed to continue unrecognized by cooximetry or arterial blood gas and untreated by methylene blue, significant morbidity and mortality can result from tissue hypoxia. This requires a heightened index of suspicion when relevant exogenous substances like local anesthetics are incorporated into therapy. We report an episode of methemoglobinemia in which clinical diagnosis was challenging given lack of cues pointing towards a diagnosis.

## 2. Case Presentation

A 27-year-old 100 kg woman presented for low anterior resection for endometriosis. The patient's history included anemia and obesity for which she previously underwent a gastric banding procedure. The surgical procedure was completed under general anesthesia with no relevant complications and the patient was transferred to the surgical intensive care unit. Recovery was complicated by an episode of ileus on postoperative day 6, necessitating nasogastric tube placement.

On day 7, the patient's oxygen saturation decreased to 82% and she reported headaches, drowsiness, and abdominal pain. The patient denied increased work of breathing but was noted to be tachypneic with a respiratory rate at 22–25 breaths per minute. Unchanged on 100% oxygen via nonrebreather mask, an arterial blood gas sample yielded chocolate brown colored blood. The blood gas result was as follows: pH 7.49, PCO_2_ 37, PO_2_ 138, HCO_3_ 28, BE +4, O_2_ saturation 98%, and methemoglobin level 47%. Upon further investigation, it was ascertained that a bottle of benzocaine spray had been left at the bedside to assist in the patient's tolerance of the nasogastric tube. The patient admitted using the spray 4 times over a 5-hour period and estimated each spray to be 2 seconds in length. The patient received 120 mg (1.2 mg/kg) IV methylene blue. Three hours later, arterial blood sampling on 27% oxygen yielded similar blood gas except for a methemoglobin level of 1.3% with resolution of prior symptoms.

## 3. Discussion

Methemoglobinemia presents with a variety of clinical features. Our patient's methemoglobin level was 47% where confusion, dyspnea, tachypnea, and tachycardia would be expected. A 30% circulating methemoglobin level is routinely cited as the critical value for initiation of methylene blue therapy (1-2 mg/kg of 1% solution) for methemoglobinemia; however, there are patients that do become symptomatic at circulating methemoglobin as low as 8% [5].

Several endogenous pathways have developed to correct for methemoglobinemia by reducing the oxidized heme group. These processes rely on the presence of reduced NADH and NADPH and maintain methemoglobin concentrations below 1% of total hemoglobin [8]. The major mechanism is reduction via cytochrome b5 reductase, accounting for 95–99% activity. Minor pathways that utilize NADPH methemoglobin reductase, reduced flavin, tetrahydropterin, cysteamine, and reduced cysteine on protein molecules are also in existence. Of note is the NADPH methemoglobin reductase pathway which has a particular affinity for dyes. It is this affinity that is exploited with the use of methylene blue in the treatment of methemoglobinemia [3, 4]. As would be expected, methemoglobin levels increase when these pathways are exhausted, when enzymes are deficient, or when methemoglobin production abnormally increases. The latter occurs when exogenous substances or their metabolites are potent oxidizers that induce excessive methemoglobin formation.

Given that methemoglobin is formed as part of a redox reaction, intuitively one can conclude that high oxidative states would predispose to the development of methemoglobinemia. This reasoning appears to hold true as septic patients have an increased risk of developing methemoglobinemia with concomitant use of benzocaine [9–11]. Other predisposing factors include baseline anemia, cancer, gastric acid suppression, and gastric mucosa inflammation [7, 9, 12].

The link between benzocaine use and methemoglobinemia has long been recognized with the FDA issuing a public advisory warning in 2006 which was later updated in 2011 advising both the public and clinicians to be judicious in their use of benzocaine following reports of 319 cases of benzocaine induced methemoglobinemia with three reported deaths [13]. Benzocaine comes in a variety of preparations including Hurricaine, Cetacaine, Exactacain, and Topex. The above patient self-administered Hurricaine spray (20% benzocaine spray (200 mg/mL)). The manufacturer's package insert states that a 3-4-second spray is equivalent to 1 mL of aerosolized 20% benzocaine. In terms of dosing, they recommend a 0.5-second spray, which may be repeated up to a maximum of four times a day. When used correctly, this technique should deliver a 30 mg dose. The manufacturer strongly advises against sprays in excess of two seconds [14]. Our patient admitted administering 4 sprays each 2 seconds in duration. This would result in a total dose of 120 mg per spray with a five-hour total dose of 480 mg. Keeping in mind that benzocaine toxicity is reported at doses as low as 100 mg, this patient received 4.8 times more in a relatively short time span.

Overuse of benzocaine is thought to be the most likely risk factor for development of benzocaine induced methemoglobinemia; however, current evidence suggests that this may not necessarily be the case. Methemoglobinemia has been noted to develop with as little as a one-second spray [15, 16]. Likely contributing to this is the fact that orientation of the canister appears to have an effect on the total administered dose. According to Khorasani et al. [17], the dose of benzocaine administered when specifically using the Hurricaine canister is highly dependent on orientation with upright positioning resulting in larger doses compared to horizontal or upside down positioning with the resultant dose per second spray varying from less than 76 mg to 212 mg [17].

Quick recognition and evaluation of the patient's complaints allow for this case to be an effective teaching tool instead of something more catastrophic. A raised index of suspicion should be met with attempts to improve oxygenation, even with assisting ventilation if necessary. Diagnosis can be made with cooximetry or methemoglobin levels and the treatment of choice is methylene blue. Adjustments to therapy are required in the event of G6PD deficiency as methylene blue is simply ineffective due to low levels of NADPH. In G6PD deficiency, the use of ascorbic acid is advocated with the caveat that it would take longer for clinical resolution to occur.

Expanding outside the realm of clinical management, cases like this demand attention towards more practical means of preventing adverse events. With the advent of electronic medical records and electronic prescribing, perhaps this case highlights the important role of medication tracking. Given that methemoglobinemia presents in a nonspecific way, tracking of benzocaine administration via barcode scanning into the electronic medication administration record would have allowed for identification of methemoglobinemia as a potential diagnosis even prior to blood gas analysis.

The FDA as discussed earlier has long recognized safety concerns with regard to the use of benzocaine. This case highlights the ease with which adverse outcomes can result from simple mistakes. Fortunately, this patient was in a monitored environment where prompt identification and treatment were possible. It is not difficult to imagine a similar clinical scenario unfolding in a patient's home resulting in significant morbidity or even mortality. This risk warrants the development of a more practical means of external control. Potential solutions would include reconfiguring benzocaine spray canisters to allow for a maximum of 100 mg only per canister. Alternatively, the bottles could be redesigned to include metered dosing for more predictable total dose actuation. However, the question still remains whether benzocaine should be available at all on the market. As discussed earlier, lidocaine, an alternative to benzocaine, is comparatively much safer. It is available as a topical aerosolized spray with an attachable atomizer for use in hard-to-reach places such as the back of the throat. These bottles also feature metered dosing for predictable dose actuation per spray. Patient safety must take precedence above all else and perhaps it is time that the use of benzocaine is reconsidered in favor of safer alternatives.

## Figures and Tables

**Table 1:** Expected signs and symptoms for a given percentage methemoglobin level assuming normal starting hemoglobin [6, 7].

Percentage methemoglobin	Expected symptoms
<10%	None
10–20%	Peripheral and central cyanosis
20–30%	Anxiety, tachycardia, lightheadedness, and headache
30–50%	Confusion, dizziness, fatigue, tachypnea, and worsening tachycardia
50–70%	Acidosis, arrhythmia, seizures, and coma
>70%	Death

## References

[1] Darling R. C., Roughton J. W. (1942). The effect of methemoglobin on the equilibrium between oxygen and hemoglobin. *The American Journal of Physiology—Legacy Content*.

[2] Kurapati S., Mehta A. C., Jain P. (2007). Benzocaine-induced methemoglobinemia. *Journal of Bronchology*.

[3] Jaffé E. R. (1981). Methemoglobin pathophysiology. *Progress in Clinical and Biological Research*.

[4] McLean S., Murphy B. P., Starmer G. A., Thomas J. (1967). Methaemoglobin formation induced by aromatic amines and amides. *Journal of Pharmacy and Pharmacology*.

[5] Guay J. (2009). Methemoglobinemia related to local anesthetics: a summary of 242 episodes. *Anesthesia & Analgesia*.

[6] Wright R. O., Lewander W. J., Woolf A. D. (1999). Methemoglobinemia: etiology, pharmacology, and clinical management. *Annals of Emergency Medicine*.

[7] Anderson C. M., Woodside K. J., Spencer T. A., Hunter G. C. (2004). Methemoglobinemia: an unusual cause of postoperative cyanosis. *Journal of Vascular Surgery*.

[8] Bloom J. C., Brandt J. T., Klaassen C. D. (2001). Toxic responses of the blood. *Casarett and Doull’s Toxicology: The Basic Science of Poisons Online*.

[9] Kane G. C., Hoehn S. M., Behrenbeck T. R., Mulvagh S. L. (2007). Benzocaine-induced methemoglobinemia based on the Mayo Clinic experience from 28 478 transesophageal echocardiograms: incidence, outcomes, and predisposing factors. *Archives of Internal Medicine*.

[10] Krafte-Jacobs B., Brilli R., Szabó C., Denenberg A., Moore L., Salzman A. L. (1997). Circulating methemoglobin and nitrite/nitrate concentrations as indicators of nitric oxide overproduction in critically ill children with septic shock. *Critical Care Medicine*.

[11] Ohashi K., Yukioka H., Hayashi M., Asada A. (1998). Elevated methemoglobin in patients with sepsis. *Acta Anaesthesiologica Scandinavica*.

[12] Novaro G. M., Aronow H. D., Militello M. A., Garcia M. J., Sabik E. M. (2003). Benzocaine-induced methemoglobinemia: experience from a high-volume transesophageal echocardiography laboratory. *Journal of the American Society of Echocardiography*.

[14] Package Insert Hurricaine topical anesthetic—benzocaine spray. http://www.pdr.net/drug-summary/hurricaine-topical-anesthetic-spray?druglabelid=2182.

[15] Guerriero S. E. (1997). Methemoglobinemia caused by topical benzocaine. *Pharmacotherapy*.

[16] O’Donohue W. J., Moss L. M., Angelillo V. A. (1980). Acute methemoglobinemia induced by topical benzocaine and lidocaine. *Archives of Internal Medicine*.

[17] Khorasani A., Candido K. D., Ghaleb A. H., Saatee S., Appavu S. K. (2001). Canister tip orientation and residual volume have significant impact on the dose of benzocaine delivered by hurricaine spray. *Anesthesia & Analgesia*.

